# Transcriptome analysis reveals the role of the PCP pathway in fipronil and endotoxin-induced lung damage

**DOI:** 10.1186/s12931-019-0986-1

**Published:** 2019-02-01

**Authors:** Arif Ahmad Pandit, Ravi Kumar Gandham, C. S. Mukhopadhyay, Ramneek Verma, R. S. Sethi

**Affiliations:** 1 Department of Animal Biotechnology, School of Animal Biotechnology, Guru Angad Dev Veterinary and Animals Sciences University, Ludhiana, Punjab 141004 India; 2 Division of Veterinary Biotechnology, ICAR-Indian Veterinary Research Institute [Deemed University], Izatnagar, Bareilly, UP, India. National Institute of Animal Biotechnology, Hyderabad, India

## Background

Around 4.6 million tonnes of pesticides are used annually and 1.8 billion people globally apply pesticides to prevent or destroy pest in agricultural settings [1–3]. The rampant pesticide usage is due to demand for increased production from the limited cultivable land. Pesticide usage becomes inevitable with a new class of pesticides introduced into the market each year to oversee the menace of pesticide resistance. Pesticides are harmful because they remain in the environment for a longer period of time with limited degradable tendency [4]. Fipronil is a broad-spectrum newer class of n-phenylpyrazole insecticide, which targets Gamma Amino Butyric Acid Receptor (GABAR) receptors as an antagonist [5]. Both humans and animals possess these receptors in the lungs making them vulnerable to its intentional damage [6]. We reported earlier that fipronil induces lung inflammation in both in vivo and in vitro models [7].

Living beings while eating the food contaminated with these micropollutants along with inhalation of airborne contaminants such as endotoxins are exposed to higher risks of pulmonary damage [8, 9]. Endotoxins are lipopolysaccharide (LPS) molecules derived from the cell membrane of many Gram-negative bacteria and are present ubiquitously in the indoor [10], occupational [11] and outdoor environment [12]. LPS interaction with various classes of pesticides modulates pulmonary responses during lung inflammation [9, 13–17]. Wnt signaling is reported to be activated during sepsis-induced lung injury by some researchers [18]. The conserved microbial structure triggers the expression of Wnt-6 and activates the Wnt/planar cell polarity (PCP) pathway that drives the polarization of macrophages to M2- phenotype in inflamed lungs [19]. The dysregulation of Wnt signaling causes the pathogenesis of chronic inflammatory diseases such as pulmonary fibrosis and interstitial pneumonia [20, 21]. Experiments on LPS induced lung injury have shown that the damage is promoted by Wnt signaling driving the Th17 cell response which culminates in the production of Il17 cytokine [22].

Wnt signaling pathways includes β-catenin dependent canonical (Wnt pathway) and β-catenin independent non-canonical (PCP pathway and Wnt/calcium pathway) pathways [23]. PCP signaling pathway essentially regulates proliferation, differentiation, apoptosis, motility, and polarization of cells [24]. Dishevelled (Dsh), an intracellular protein, mediates both Wnt/β-catenin and PCP pathways [25, 26]. Dsh or DvL (mammalian homologue of Drosophila Dishevelled) is a modular protein which acts as transducer during PCP signaling. It is recruited from the cytoplasm of the cell to its plasma membrane by PCP receptor activating the pathway [27]. Overexpression of disheveled protein activates the Mitogen-Activated Protein Kinase (Mapk) c-Jun N-terminal Kinase (Jnk) pathway [28, 29] which functions downstream of dishevelled to mediate the PCP pathway [30, 31]. PCP pathway can exert both pro-inflammatory and anti-inflammatory functions in inflammation [32–34].

Animal studies have shown that exposure to respiratory irritants activates the pulmonary immune system which contributes to the release of various cytokines like interleukin *Il4* and *Il17* [35, 36]. The pathophysiological and repair processes occurring during inflammation are mainly mediated by pro-inflammatory and anti-inflammatory cytokines. Il4 is an anti-inflammatory cytokine [37] that increases the expression of non-canonical Wnt proteins during infection or inflammation [38]. Murine peritoneal macrophages treated with Il4 have been shown to overexpress Wnt proteins confirming its role in mucosal repair process [39]. Il17 is a pro-inflammatory cytokine and plays important role in the host defence against multiple pathogens by controlling the recruitment of neutrophils and other immune cells to the infection site [40]. Recently HMGB1-TLR4-IL23-IL17A axis has been reported to play an important role in the pathogenesis of paraquat-induced lung injury [41]. While pesticide-induced lung injury is inevitable, it is important to contemplate the mitigation strategies where computationally we can obtain the location and the magnitude of the inflammation using spirometry data and use appropriate drugs to control it [42–44].

While non-canonical Wnt ligands exert proinflammatory action on macrophages [32] and endothelial cells [45]; the role of PCP signaling remains poorly understood during lung damage. Hence, the study was aimed to elucidate the molecular mechanisms underlying the lung injury induced by fipronil alone or in combination with endotoxin. We hypothesized that fipronil induced lung inflammation is mediated by the PCP pathway and employed a mouse model to test the hypothesis by using a microarray approach along with Ingenuity Pathway Analysis [IPA©] a bioinformatics tool. The data show the PCP pathway as a top dysregulated pathway along with increased expression of *Wnt-6* and downstream production of Il17c and Il4 during fipronil induced lung inflammation.

## Methods

### In vivo experiments

The experimental protocols were dually approved by Institutional Animal Ethics Committee, Guru Angad Dev Veterinary and Animal Sciences University, Ludhiana as per the guidelines from the committee for control and supervision of experiments on animals (CPCSEA). Forty-two healthy male albino mice aged 6–8 weeks were purchased from the disease-free small animal house, Lala Lajpat Rai University of Veterinary and Animal Sciences, Hisar Haryana. The mice were maintained under controlled conditions in polypropylene cages with 12 h. light and 12 h. dark cycle at small animal housing hall, GADVASU, Ludhiana. The animals were given synthetic pelleted diet and water ad libitum. The mice were acclimatized for one week prior to the start of the experiment.

### Experimental design

Animals were weighed and randomly divided into three groups i.e. two treatments and one control (*n* = 14 in each group). LD_50_ of fipronil in male mice is 95 mg/Kg [46]. Treatment groups were given 1/10th of LD_50_ (9.5 mg/kg) and 1/20th of LD_50_ (4.75 mg/kg) of fipronil dissolved in corn oil per animal per day orally for 90 days, respectively. We selected two different doses of fipronil (9.5 mg/kg and 4.75 mg/kg) based on the previous report [47] that these doses are unlikely to cause any known toxic effects. The control group was given corn oil orally for 90 days.

Immediately after completion of the treatment period, seven animals from each group were anesthetized with 1/10th of the actual dose of xylazine-ketamine combination (Xylazine @ 0.5 ml; 20 mg/ml mixed with Ketamine @ 2 ml; 50 mg/ml) intraperitoneally. After anesthesia, *E. coli* LPS was administered @ 80 μg/animal via an intranasal route as described earlier [9]. The remaining seven animals from each group were given 80 μl of normal saline solution (NSS) per mouse via the intranasal route. The animals were euthanized after 9 h of LPS/NSS exposure with a full dose of the xylazine-ketamine combination (0.1 μl / 10 g of body weight).

### Tissue collection

Bronchioalveolar lavage (BAL) fluid was collected from left lung and subjected to total leucocyte counts (TLC) and differential leucocyte counts (DLC) on the same day as previously described [9]. The right lung was ligated and collected aseptically in RNase free 1.5 ml centrifuge tube containing 1 ml RNAlater (Ambion, Austin, TX, USA) and stored at − 80 °C for RNA isolation (transcriptome analysis). Left lung tissues samples were collected in paraformaldehyde solution for histopathology and immunohistochemistry.

### Hematoxylin and eosin staining

The left lung was fixed in-situ and stored in paraformaldehyde solution at 4 °C for 12 h and processed to obtain 5 μm thick paraffin sections. The sections were stained with hematoxylin and eosin for histopathological analysis. The lung sections were evaluated in a blinded fashion to grade the degree of lung injury, using a previously described scoring system [48] with slight modifications. The pathological features viz. peribronchial infiltration, perivascular infiltration, capillary congestion, increase in perivascular space, thickening of alveolar lining and interalveolar edema were evaluated. Each feature was scored from 0 to 3 based on its absence (0) or presence to a mild (1), moderate (2), or severe (3) degree to obtain cumulative total histology score (THS).

### Microarray gene expression and analysis

About 50 mg of lung tissue was used from all the animals to isolate RNA using the Trizol method (Ambion, Life Technologies, USA) following the manufacturer’s instructions. The purity and concentration of total RNA extracted were checked using the Nanodrop spectrophotometer (Thermo Fisher). The quality check of the isolated RNA was performed in Agilent 2100 Bioanalyzer as per manufacturer’s protocol using the Agilent RNA 6000 Nano Kit. RNA samples with an RNA Integrity Number (RIN) > 7 were used for microarray hybridization.

Total RNA (100 ng) was labeled with Low Input Quick Amp WT Labeling Kit as per manufacturer’s instructions. RNA samples from three mice in each group were pooled into two samples (biological replicates) and One-color microarray-based exon analysis was performed in duplicates (technical replicates) using two mouse microarray slides (8x60K: Agilent—028005). The quality check of the labeled cRNA was performed using NanoDrop and the yield and specific activity were estimated. After generating the microarray scan images, the signal intensities were extracted using Feature Extraction software version 10.7.3. The data generated were analyzed using GeneSpring software version 14.9 (Agilent Technologies) to identify the differentially expressed genes (DEGs) (*P* ≤ 0.05). Hierarchical clustering of the DEGs was done to observe and interpret the data.

### Functional annotation

We conducted Gene Ontology (GO) enrichment analysis of the DEGs and uniquely expressed genes to investigate the biological processes that are enriched in the experimental groups. Three groups of GO categories i.e. biological process, cellular component, and molecular function were separately analyzed by Agilent Gene Spring software version 14.9. Genes with *p* < 0.05 were selected and tested against the background set of all genes with GO annotations.

### Downstream comparison analysis by ingenuity pathway analysis (IPA)

Gene lists containing gene identifiers (probe set IDs), and corresponding expression values (fold change) were uploaded to Ingenuity Pathway Analysis (IPA), a web-based bioinformatics tool (Ingenuity® Systems https://www.qiagenbioinformatics.com/products/ingenuity-pathway-analysis/). Each gene identifier was mapped to its corresponding gene object in the Ingenuity Pathways Knowledge Base. The data sources from ingenuity expert findings were used to run the “Comparison Analysis” to identify the significant (*p* < 0.05), activated (Z score > 1.5) and inactivated (Z score < − 1.5) canonical pathways.

### Quantitative real-time PCR

Quantitative Real-time PCR was carried out on the same lung samples that were used for the microarray to validate the microarray data on the expression of *Wnt-6*, *Mapk8*, *Il4*, and *Il17c*. The total RNA was isolated from all the samples and concentration of total RNA varied between 1500 and 3800 ng/μl in different samples. The amount of total RNA used for cDNA synthesis was adjusted to 400 ng/μl for each sample. Total RNA was reversed transcribed into cDNA using a First-strand cDNA synthesis kit (Thermo Scientific, USA) according to the manufacturer’s instruction. The real-time PCR reaction was performed in duplicate by using Syber green chemistry with β- actin as an endogenous control. The primer sequences used for the selected genes were earlier reported for *Wnt6* [19], *Mapk8* [49], *Il17c* [50], *Il4* [51] and Actb [50]. The relative expression of each sample was calculated using the ΔΔCT method [52].

### Immunohistochemistry

The immunohistochemistry was carried out as described earlier [53]. Briefly, the sections were first deparaffinized, dehydrated, incubated with 3% H_2_O_2_ for 20 min to quench endogenous peroxidase and followed by boiling in Tris-borate EDTA and 1X PBS for antigen retrieval. The slides were incubated in a dark chamber with 1% BSA. The sections were stained with primary antibodies against mouse Wnt6 (rabbit polyclonal Wnt6; E-AB-17612; dilution 1:100), Mapk8 (Jnk1) (rabbit polyclonal Jnk1/2/3; E-AB-20915; dilution 1:20), Il4 (rabbit polyclonal Il4; E-AB-33415; dilution 1:20) and Il17c (rabbit polyclonal Il17c; E-AB-13324; dilution 1:75) followed by appropriate horseradish peroxidase (HRP)-conjugated secondary antibody (Polyclonal goat anti-rabbit; BA-1000; dilution 1:400; Vector Laboratories). The reaction was visualized using a color development kit (SK4600, Vector Laboratories, USA). The sections were counterstained with methyl green. Controls consisted of staining without primary antibody or secondary antibody or both.

### Grading for immunohistochemistry

Lung sections from all the animals were used for grading of immunohistochemical staining intensity and quantification of the number of immunopositive Wnt6, Mapk8, Il4 and Il17c cells in those areas that were fully cross-sectioned. The cells were counted in 10 fields/section manually in an area of 0.2 mm^2^ under the 40× objective lens of the microscope so as to maintain the uniformity as described earlier [54]. Five animals from each group were randomly selected for quantification of these cells. The evaluator was blinded to the identity of treatment groups.

### Statistical analysis

Data from TLC, DLC, histopathology, immunohistochemistry, and ΔCT values were presented as mean ± SEM. Further, data were subjected to analysis of variance (ANOVA) followed by Tukey’s posthoc test, using GraphPad Prism software (evaluation version).

## Results

### TLC and DLC counts in BAL fluid

**Fig. 1:**
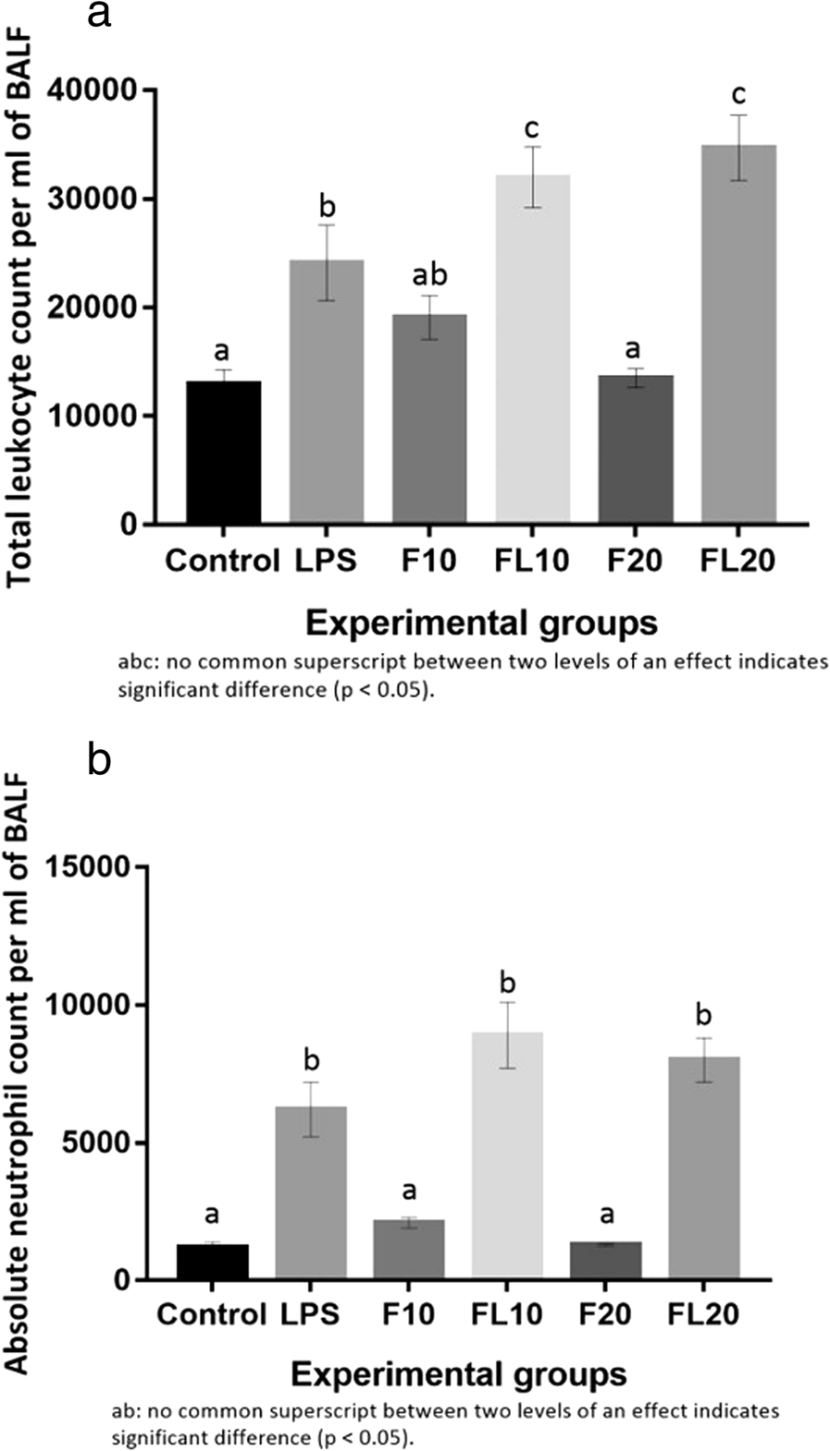
Total leucocyte (**a**) and absolute neutrophil count (**b**) per ml of BAL fluid in LPS (CL), high dose (1/10th of LD_50_) of fipronil alone (F10) or in combination with LPS (FL10), low dose (1/20th of LD_50_) of fipronil alone (F20) or in combination with LPS (FL20) group

LPS exposure increased (*p* < 0.05) the TLC of BAL fluid along with neutrophilia, however, fipronil at both doses did not alter the TLC or absolute neutrophil counts (Fig. 1). Further, low and a high dose of fipronil along with LPS increased (*p* < 0.05) TLC as compared to LPS or individual fipronil group (Fig. 1).

### Confirmation of inflammation by histopathological observations

**Fig. 2:**
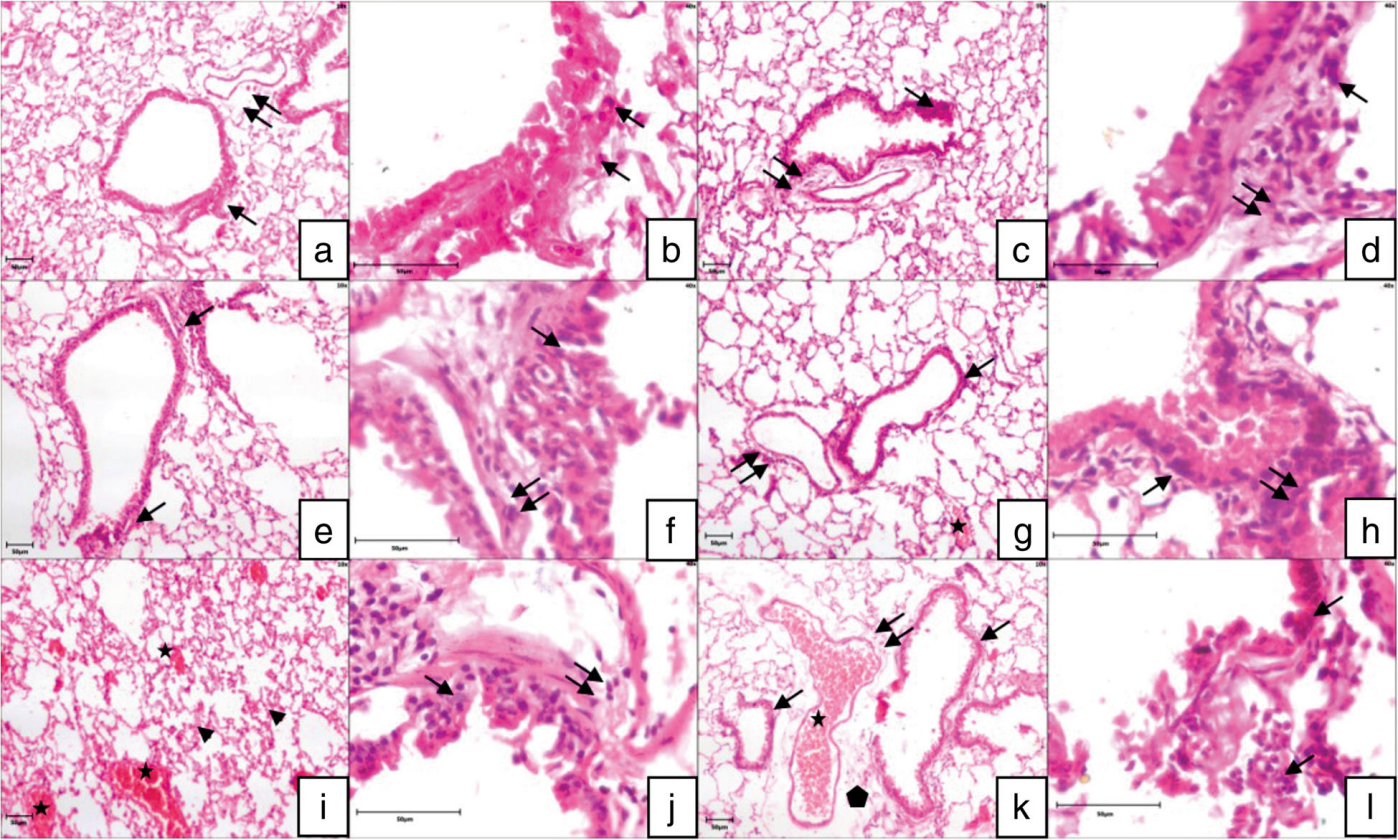
H&E staining: Lung sections of control mice (**a**, **b**) showed normal architecture of the airways epithelium (arrow) and alveolar septa (double arrow). Peribronchial (arrow) and perivascular infiltration (double arrow), congestion of blood vessels (star) and increase in perivascular space (polygon) in LPS (**c**, **d**), high dose (1/10th of LD_50_) of fipronil alone (**e**, **f**) or in combination with LPS (**g**, **h**), low dose (1/20th of LD_50_) of fipronil alone (**i**, **j**) or in combination with LPS (**k**, **l**) group. Original magnification: 2a,c,e,g,i,k: 10X; 2b,d,f,h,j,l: 40X

Hematoxylin and eosin stained lung sections showed normal histoarchitecture of the lung in the control group (Fig. 2a, b). LPS and both doses of fipronil individually or in combination with LPS resulted in lung inflammation characterized by peribronchial and perivascular infiltration of mononuclear inflammatory cells, congestion in blood vessels and increase (*p* < 0.05) in the total histology score in all groups compared to the control (Fig. 2c-l).

### Differentially expressed genes (DEGs) and functional analysis

**Fig. 3:**
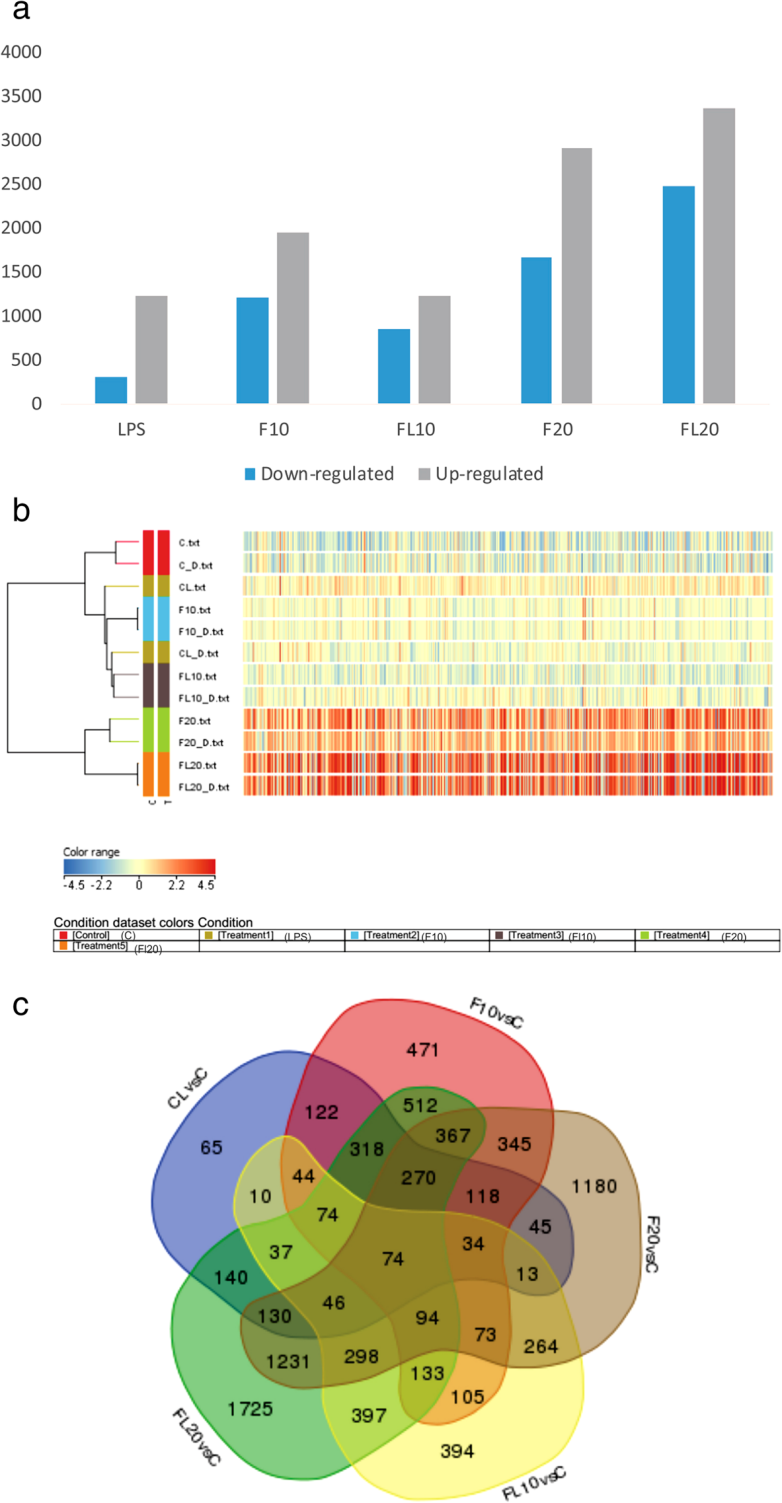
**a**) Global view of differentially expressed genes (DEG’s) **b**) Hierarchical clustering of samples based on DEGs with at least 2-fold change and controlled by false discovery rate of 0.1, as inferred from treated versus normal samples. Red indicates upregulation while blue indicates downregulation. In the sample clustering dendrogram, red indicates control samples, dark green indicates (LPS treated), blue indicates (fipronil at high dose 1/10th of LD_50_), brown indicates (fipronil at high dose followed by LPS), light green indicates (fipronil at low 1/20th of LD_50_ dose), while orange indicates (fipronil at low dose followed by LPS) samples. **c**) Venn diagram showing the overlap of DEG’s involved across LPS (CL), high dose of fipronil alone (F10) or in combination with LPS (FL10), low dose of fipronil alone (F20) or in combination with LPS (FL20) group

A total of 5847 genes were differentially expressed (*p* < 0.05; fold change > **±**1.5) following exposure to LPS and high (9.5 mg/kg) and low (4.75 mg/kg) dose of fipronil. After exposure to LPS, 1231 genes were up-regulated and 309 genes were down-regulated (Fig. 3a). Treatment with a high dose of fipronil caused the up-regulation of 1942 genes and down-regulation of 1212 while the same dose combined with LPS up-regulated 1236 genes and downregulated 854 genes. A low dose of fipronil up-regulated 2912 genes and down-regulated 1670 genes. The same dose in combination with LPS up-regulated 3368 genes and down-regulated 2478 genes. Differential expression of the genes included in Fig. 3a was visualized by a heat map obtained by hierarchical clustering (HCL), which generates a tree (dendrogram) to group similar objects together (Fig. 3b). Through gene expression profiles, 74 genes (66 up-regulated and 9 down-regulated) were commonly expressed in all the treatment groups as compared to control (Fig. 3c). The relative expression levels of these genes are illustrated as a heat map (Additional file 1: Figure S1).

Gene Ontology (GO) analysis (*p*-value< 0.05) of DEGs enriched the genes involved in various biological processes viz. transmembrane signaling activity, G-protein coupled receptor signaling pathway and molecular transducer activity. Further, 46% of DEGs were enriched in transmembrane signaling activity while 21 and 29% of DEG’s were involved in various molecular functions and cellular component activities, respectively (Additional file 2: Table S1).

### Ingenuity pathway analysis of differentially expressed genes

IPA analysis of DEGs revealed that PCP pathway was the top canonical pathways along with HMGB1 signaling, eicosanoid signaling, basal cell carcinoma signaling, CD27 signaling in lymphocytes following exposure to both doses of fipronil with or without LPS (Table 1). PCP pathway showed higher enrichment in fipronil low dose group alone or in combination with LPS compared to the other groups. PCP pathways generated with help of IPA software for all groups are presented in (Additional file 1: Figure S2-S4).

**Table 1:** Top canonical pathways following exposure to fipronil with or without LPS

Canonical pathway	-log (*p*-value)				
	CL	F10	F20	FL10	FL20
PCP pathway	0.40	0.08	0.50	1.20	2.50
Hmgb1 signaling	0.65	0.95	1.50	0.75	1.54
Eicosanoid signaling	1.71	0.63	1.39	0.51	1.52
Basal cell carcinoma signaling	0.83	0.49	0.01	0.43	1.75
CD27 Signaling in Lymphocytes	0.02	0.48	1.54	0.78	1.29

The -log (*p*-value) refers to the level of enrichment

CL: LPS treated, F10: Fipronil at high dose, FL10: fipronil at high dose followed by LPS, F20: Fipronil at a low dose and FL20: Fipronil at low dose followed by LPS

### Validation of microarray data by real-time RT-PCR and immunohistochemistry

#### Wnt6

Microarray analysis data revealed that exposure to LPS and individual high and a low dose of fipronil increased (*p* < 0.05) *Wnt6* mRNA expression by 1.15, 1.61 and 2.9 folds, respectively. While there was only 0.48 fold increase in the *Wnt6* expression following co-exposure to LPS and a high dose of fipronil, the low dose in combination with LPS increased the expression by 4.23 fold. The validation of *Wnt6* mRNA expression analysis by real-time PCR was in concordance with microarray data (Fig. 6a).

**Fig. 4:**
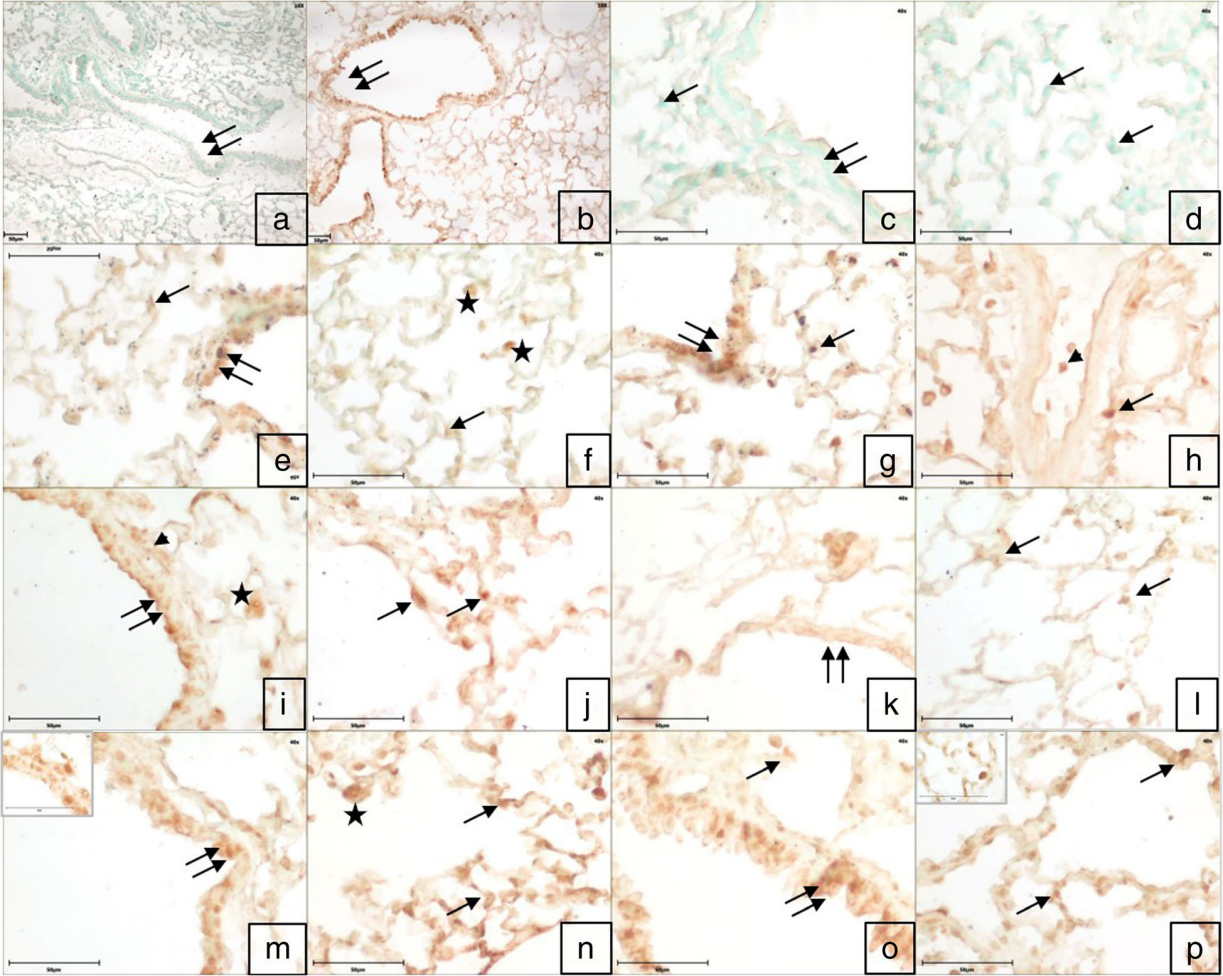
Immunohistochemistry for expression of *Wnt6*: Lung section stained without primary antibody (**a**, **c**, **d**) does not show any color development in airways epithelium (double arrow), alveolar septal cells (arrow) and alveolar macrophages (star). Immunopositive *Wnt6* reactivity in airways epithelium (double arrow), alveolar septa (arrow) and alveolar macrophages (star) in the control group (**e**, **f**). Immunopositive *Wnt6* reactivity in airways epithelium (double arrow), alveolar septa cells (arrow), alveolar macrophages (star) and infiltrating cells (arrowhead) in LPS (**g**, **h**) high dose (1/10th of LD_50_) of fipronil alone (**i**, **j**) or in combination with LPS (**k**, **l**), low dose (1/20th of LD_50_) of fipronil alone (**b**, **m**, **n**) or in combination with LPS (**o**, **p**) group. Original magnification **a**, **b**: 10X and **c**-**p**: 40X

**Fig. 5:**
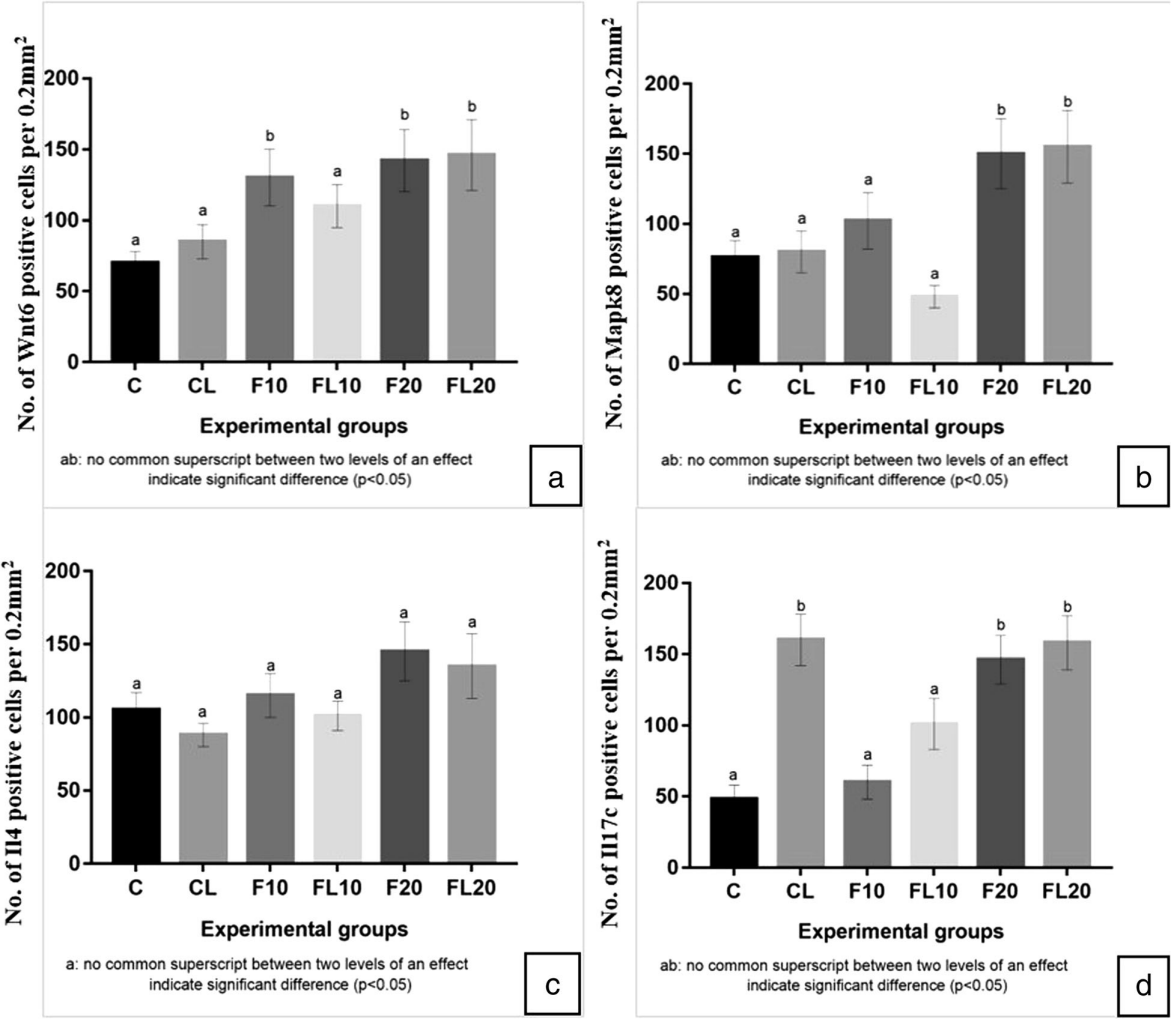
Quantification of immunopositive cells **a**) *Wnt6*, **b**) *Mapk8*, **c**) *Il4* and **d**) *Il17c* in control (**C**), LPS (CL), high dose (1/10th of LD_50_) of fipronil alone (F10) or in combination with LPS (FL10) and low dose (1/20th of LD_50_) of fipronil alone (F20) or in combination with LPS (FL20) group

The omission of the primary antibody or both primary and secondary antibodies resulted in a lack of staining in the tissue sections (Fig. 4). Immunopositive Wnt6 reactivity was observed in the airways epithelial cells, alveolar septal cells and occasionally macrophages in control. Mild Wnt6 positive immune reactivity was also localized in the endothelial cells and some infiltrating cells in the blood vessels following LPS challenge and in large septal cells following exposure to high dose of Fipronil (Fig. 4g, h). Fipronil at low dose with or without LPS showed a strong immunopositive Wnt6 reactivity in bronchial epithelium and alveolar septa (Fig. 4m-p). There was an increase (*p* < 0.05) in the number of immunopositive Wnt6 cells following exposure to both doses of fipronil and a low dose of fipronil in combination with LPS compared to control group (Fig. 5a).

#### Mapk8

**Fig. 6:**
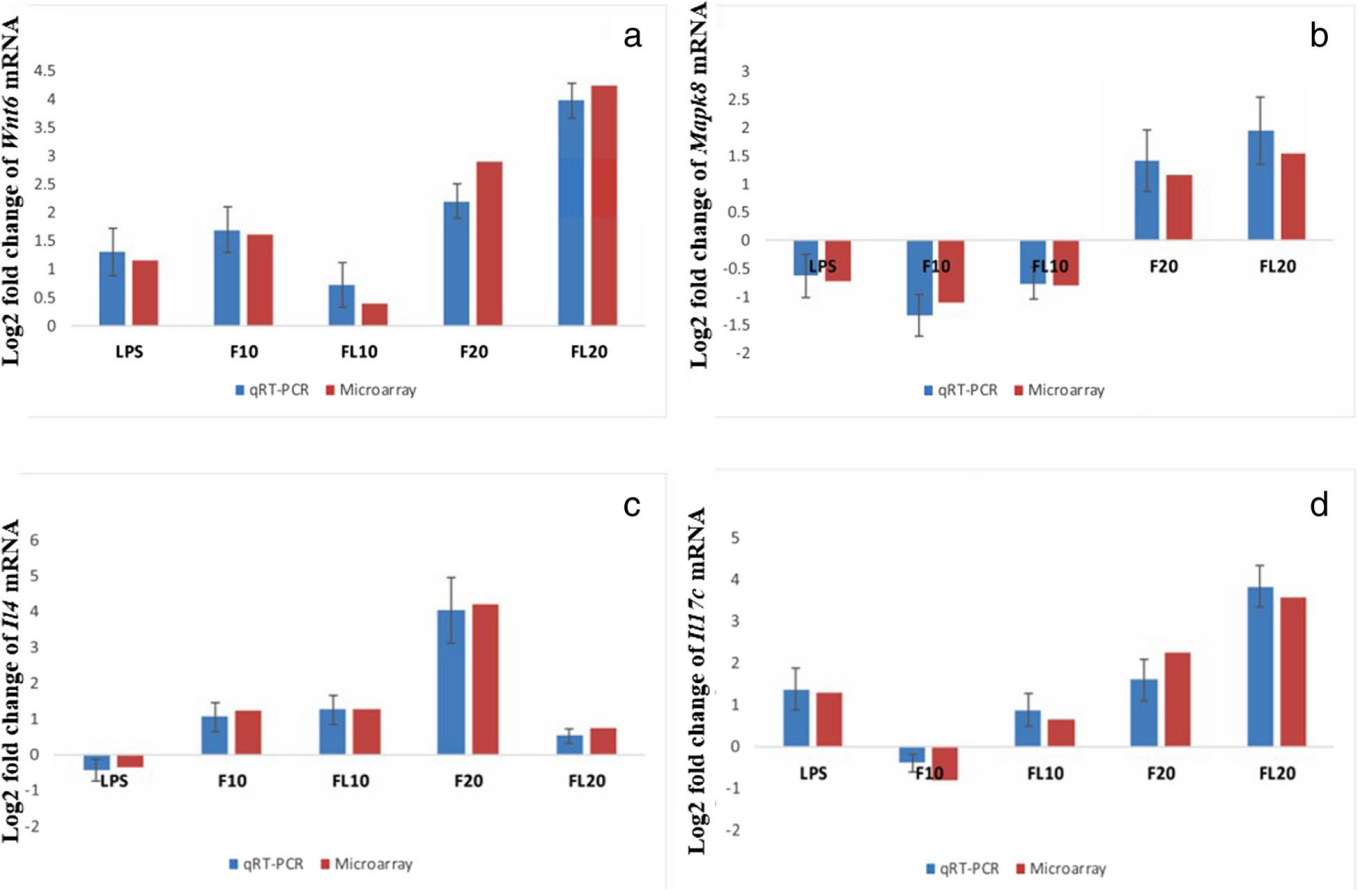
Fold change concordance of *Wnt6* (**a**), *Mapk8* (**b**), *Il4* (**c**) and *Il17c* (**d**) by qPCR. The expression levels represent log_2_ fold change values calculated from the normalized signal intensity values using corn oil/NSS placebo expression data as control. Gene expression in each sample was normalized against the expression of Actb gene

Microarray data depicted 0.72, 1.1 and 0.78 fold decrease (*p* < 0.05) in *Mapk8* mRNA expression following exposure to LPS alone, high dose of fipronil alone or in combination with LPS, respectively. However, low dose of fipronil alone or in combination with LPS increased (*p* < 0.05) the expression of *Mapk8* by 1.2 and 1.55 fold, respectively. The validation of *Mapk8* mRNA expression by real-time PCR was in concordance with microarray data (Fig. 6b).

**Fig. 7:**
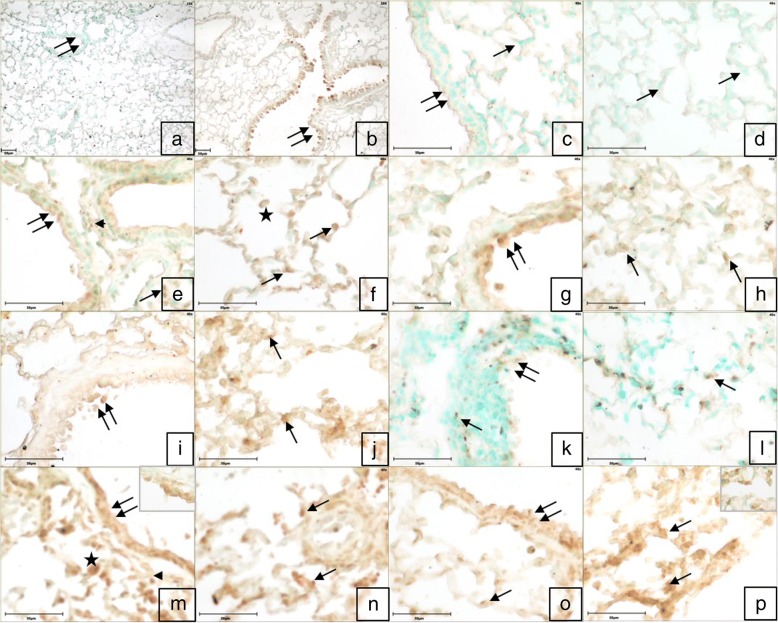
Immunohistochemistry for expression of *Mapk8*. Lung section stained without primary antibody (**a**, **c**, **d**) showed no color development in airways epithelium (double arrow) and alveolar septal cells (arrow). Immunopositive reactivity in airways epithelium (double arrow) and alveolar septal cells (arrow), alveolar macrophages (star) and infiltrating cells (arrowhead) in control (**e**, **f**), LPS (**g**, **h**), high dose (1/10th of LD_50_) of fipronil alone (**i**, **j**) or in combination with LPS (**k**, **l**), low dose 1/20th of LD_50_) of fipronil alone (b,m,n) or in combination with LPS (**o**, **p**) group. Original magnification **a**, **b**: 10X and **c**-**p**: 40X

Similarly, low dose of fipronil with or without LPS resulted in a strong Mapk8 immunopositive reactivity in the airways epithelium, alveolar wall and infiltrating cells around airways epithelium (Fig. 7) along with increase (*p* < 0.05) in the number of immunopositive Mapk8 cells compared to control group (Fig. 5b). However, a high dose of fipronil (9.5 mg kg^− 1^) showed a moderate immunopositive reactivity in the bronchial epithelium and alveolar septal cells (Fig. 7).

#### Il4

Microarray data indicated 0.37 fold decrease (*p* < 0.05) in *Il4* mRNA expression following LPS challenge. However, a high and low dose of fipronil increased (*p* < 0.05) the expression by 1.22 and 4.2 fold, respectively. Co-exposure of LPS and a high dose of fipronil did not alter the expression (1.28 folds) as compared to an individual high dose of fipronil whereas LPS along with a low dose of fipronil downregulated the expression and limited the increase to 0.75 fold only. The validation of *Il4* mRNA expression by real-time PCR was in concordance with microarray data (Fig. 6c).

**Fig. 8:**
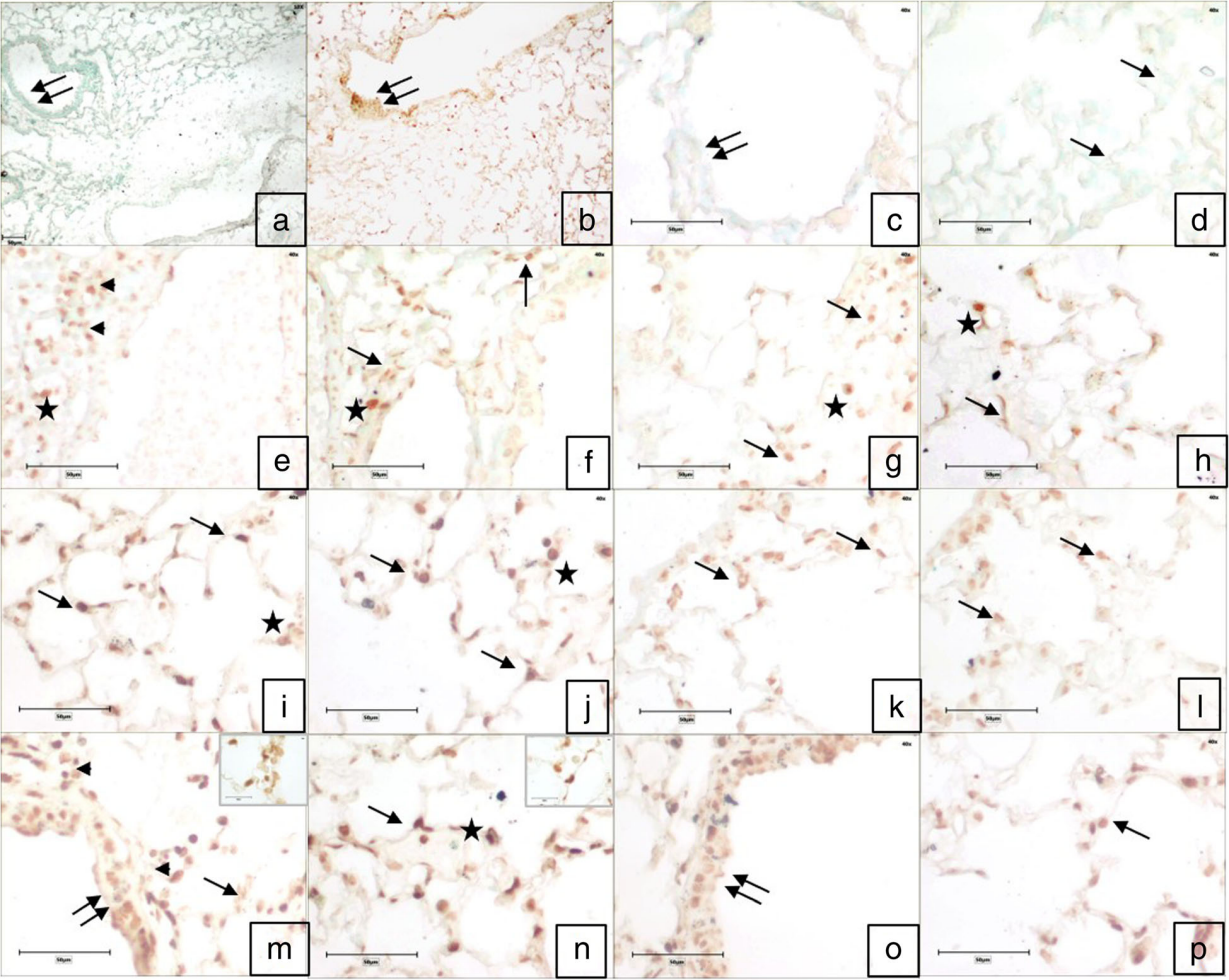
Lung section stained with *Il4* primary antibody. Control sections without primary antibody (**a**, **c**, **d**) resulted in no color development in airways epithelium (double arrow) and alveolar septal cells (arrow). Immunopositive reactivity in airways epithelium (double arrow) and alveolar septal cells (arrow), alveolar macrophages (star) and infiltrating cells (arrowhead) in control (**e**, **f**), LPS (**g**, **h**), high dose (1/10th of LD_50_) of fipronil alone (**i**, **j**) or in combination with LPS (**k**, **l**), low dose (1/20th of LD_50_) of fipronil alone (**b**, **m**, **n**) or in combination with LPS (**o**, **p**) group. Original magnification **a**, **b**: 10X and **c**-**p**: 40X

There was mild to the moderately Il4 immunopositive reactivity in the infiltrating cells and alveolar septal cells of the control group (Fig. 8e, f). A higher dose of fipronil (9.5 mg kg^− 1^) with or without LPS showed moderate reactivity of Il4 in the alveolar septal cells along with macrophages (Fig. 8k-l). A low dose of fipronil (4.75 mg kg^− 1^) with or without LPS caused a strong Il4 immunopositive reactivity in alveolar epithelial cells, alveolar macrophages and infiltrating cells along the airways. However, there was no change in the number of immunopositive Il4 cells in any treatment group compared to control (Fig. 5c).

#### Il17c

Microarray data analysis revealed that LPS increased (*p* < 0.05) *Il17c* mRNA expression by 1.28 folds, however high dose of fipronil decreased (*p* < 0.05) the expression by 0.8 folds. There was 2.25 folds increase (*p* < 0.05) in the expression of *Il17c* mRNA following exposure to low dose of fipronil. LPS combination with a high and low dose of fipronil led to an increase (*p* < 0.05) in *Il17c* expression by 0.65 and 3.55 folds, respectively. The validation of *Il17c* mRNA expression by real-time PCR was in concordance with microarray data (Fig. 6d).

**Fig. 9:**
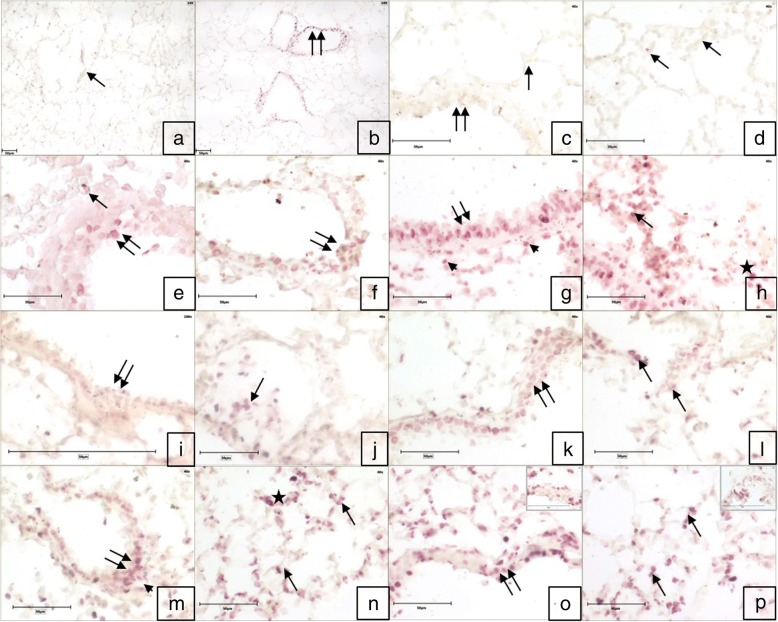
Lung section stained with *Il17c* primary antibody. Control sections without primary antibody (**a**, **c**, **d**) resulted in no color development in airways epithelium (double arrow) and Immunopositive reactivity in airways epithelium (double arrow) and alveolar septal cells (arrow), alveolar macrophages (star) and infiltrating cells (arrowhead) in control (**e**, **f**), LPS (**g**, **h**), high dose (1/10th of LD_50_) of fipronil alone (**i**, **j**) or in combination with LPS (**k**, **l**), low dose (1/20th of LD_50_) of fipronil alone (**b**, **m**, **n**) or in combination with LPS (**o**, **p**) group. Alveolar septal cells (arrow) and infiltrating cells (arrowhead). Original magnification **a**, **b**: 10X and **c**-**p**: 40X

Control group of mice showed a mild immunopositive Il17c reactivity in airways epithelia and cells lining alveolus (Fig. 9e, f). LPS challenge increased (*p* < 0.05) the number of Il17c immunopositive cells (Fig. 5d) and showed strong immunopositive reactivity in alveolar epithelial and septal cells along with some infiltrating cells (Fig. 9g, h). High dose of fipronil (9.5 mg kg^− 1^) showed a mild increase in Il17c expression (Fig. 9i, j) and the same dose in combination with LPS resulted mild to the moderate Il17c immunopositive reactivity (Fig. 9k, l). Fipronil at a low dose (4.75 mg kg^− 1^) with or without LPS increased (*p* < 0.05) the number of Il17c immunopositive cells as compared to control group (Fig. 5d) and showed a strong immunopositive reaction in the alveolar epithelial cells, alveolar macrophages and infiltrating cells (Fig. 9m-p).

## Discussion

The study aimed to elucidate the molecular mechanisms underlying the lung damage induced by fipronil alone or in combination with endotoxin. We report the first data on the PCP pathway mediated lung inflammation following oral administration of fipronil. PCP pathway was the top dysregulated pathway along with increased expression of *Wnt6* and downstream production of various cytokines during fipronil induced lung inflammation.

BAL fluid cytology along with histopathology was recorded to characterize the lung inflammation. LPS increased (*p* < 0.05) the TLC and absolute neutrophil count of the BAL fluid. LPS is known to induce lung inflammation characterized by increased TLC and neutrophils as neutrophils are attracted to the alveolar space owing to the increased epithelial permeability during lung inflammation [55]. LPS induced an increase in the cell count peaks at almost 8 h [56] to 24 h [57] of LPS instillation. We recorded similar observation after 9 h of intranasal LPS exposure which validate our mouse model of lung inflammation. Interestingly, both doses of fipronil did not alter the TLC and neutrophil count, however, when combined with LPS there was an increase in both the parameters. We have earlier reported that endotoxins/LPS interaction with pesticides like lindane [13] and imidacloprid [9] alters the of BAL fluid cytology. The data suggest that the change in BAL fluid cytology is LPS driven only.

High and low doses of fipronil alone or in combination with LPS resulted in lung inflammation characterized by peribronchial and perivascular infiltration of mononuclear inflammatory cells, congestion in blood vessels and increase (*p* < 0.05) in the total histology score in all groups compared to the control. Recently our lab showed that acute exposure to fipronil [7] or chronic oral exposure to lindane [13], chlorpyriphos [58] and indoxacarb [59] alters the lung histomorphology. Other reports also indicate lung damage following exposure to organophosphate [60], malathion [61] and chlorpyriphos [62]. The histopathological data taken together suggest both doses of fipronil resulted in lung inflammation and damage was more severe when combined with LPS.

Whole genome profiling by means of microarray transcriptome analysis has grown as an exclusive approach to identify potential candidate genes involved in the pathological processes. Here in we report the first systematic analysis to identify the underlying mechanisms involved in the fipronil induced lung inflammation. Low dose (4.75 mg kg^− 1^) of fipronil alone or with LPS highly altered transcriptome profile as compared to high dose (9.5 mg kg^− 1^). We found 5847 DEG’s among all the groups which were enriched in transmembrane signaling activity (46%) and involved in various molecular functions (21%) and cellular component activities (29%). The data suggest that fipronil alters the signal transduction and transmembrane activity and such alterations disrupt controlled cell growth and division, cell death, and cell motility that fuels cancer progression [63].

Comparison analysis using the IPA tool revealed PCP pathway as the top enriched canonical pathway suggesting the involvement of the PCP pathway in the etiology and progress of the fipronil induced lung damage. Low dose group of fipronil in combination with LPS showed significant enrichment of the PCP pathway compared to the other groups. Wnt/PCP signaling pathway is involved in cell proliferation, stem cell maintenance, cell migration, survival, and cell fate determination [64] and in various diseases including cancer [65]. Wnt signaling network, besides its role in carcinogenesis, is also found to be operative at the interface between innate and adaptive immunity [38]. Wnt proteins like Wnt-6 initiate the PCP pathway by binding to their receptors on the cell membrane [66]. In our study macrophages along with alveolar epithelial cells and occasional infiltrating cells showed Wnt-6 immunopositive reactivity. Macrophages are the primary source of *Wnt6* in the lungs [67]. During an inflammatory process, leukocytes drift across vascular endothelium [68] and Wnt/PCP pathway activation are involved in the low-level migration of monocytes which subsequently becomes the source of Wnt proteins [69]. A low dose of fipronil alone significantly increased (2.9 fold) Wnt-6 expression and the increase was more pronounced (4.23 folds) in combination with LPS. The data suggest that a lower dose of fipronil alone or along with LPS is more proactive in altering the transcriptome profile of the lungs by activating the PCP/Wnt pathway during fipronil induced lung inflammation.

The PCP works in conjunction with Jnk signalling to cause downstream regulation of target genes [30]. *Mapk8* or *Jnk-1* is the important member of Jnk family of kinases [70] and gets activated via Wnt/PCP receptor signalling following cell exposure to a variety of biotic or abiotic stress events, such as infection, inflammation, oxidative stress, DNA damage, osmotic stress, or cytoskeletal changes [71]. Activation also occurs through the release of cytokines such as *Il17* [72]. *Mapk8* is expressed by alveolar macrophages, epithelial cells, vascular endothelial cells and lymphocytes of the lung [73]. Unlike high dose, low dose of fipronil with or without LPS resulted in an increase in the expression of Mapk8 and resulted in the strong immunopositive reaction in the airways epithelium, alveolar wall and infiltrating cells around airways epithelium. *Mapk8* plays a central role in apoptosis and cellular stress responses and alters lung remodeling after an injury [74]. The data taken together suggest that fipronil at low dose with or without LPS activates *Mapk8* via PCP signaling. The activation status could have been further verified by checking the phosphorylation of Jnk1 which adds a drawback to our study.

Many alveolar cells particularly macrophages increase the production of pro- or anti-inflammatory cytokines in response to an external agent via Wnt/PCP signaling and Jnk pathways depending on the cellular context, the type of insult, and the cytokine environment [75]. *Il4* is an important anti-inflammatory cytokine that causes the differentiation of naive helper T cells (Th0 cells) to Th2 cells to regulate the cell proliferation, apoptosis and gene expression of many cell types like macrophages, fibroblasts, endothelial and epithelial cells [37]. *Il4* levels tend to peak after 12–24 h of cell stimulation [76] which could be the reason that 9 h of LPS challenge did not alter *Il4* mRNA expression in the present study. However, fipronil at lower dose increased its production by 4.2-fold along with strong Il4 immunopositive reactivity in the alveolar macrophages. *Il4* increases the expression of non-canonical Wnt proteins during infection or inflammation [38]. The data taken together along with Wnt-6 expression suggest that alveolar macrophages might have increased the production of either *Il4* by Wnt/PCP signaling or *Wnt6* (parent Wnt ligand) via positive feedback loop during fipronil induced lung damage.

The airways epithelial cells produce proinflammatory cytokines including *Il17c* along with growth factors, and chemokines that draw inflammatory cells into the airways during lung inflammation [77]. Functionally *Il17c* is a distinct member of the Il17 family which causes the cells to release TNF-α, Il8, Il1α/β, Il1F5, Il1F9, Il6, Il19 and other proinflammatory mediators [78]. Interestingly, a high dose of fipronil decreased the expression of *Il17c* and showed a mild Il17c immunopositive reactivity in alveolar epithelial and septal cells. *Il17c* deficiency reduces the recruitment of leukocyte, macrophage, and neutrophil due to defective *Il17c*-mediated Il17A+ T cell recruitment [79]. However, low dose of fipronil increased the expression of *Il17c*, the number of *Il17c* immunopositive cells along with a strong immunopositive reaction in the alveolar epithelial cells, alveolar macrophages and infiltrating cells as compared to control group. Further, LPS alone or in combination with a low or high dose of fipronil also increased the expression of *Il17c*. *Il17c* along with Il17A increases the recruitment of additional myeloid cells [79, 80]. The study does not explain the mechanism behind increased *Il17c* expression and activation of Wnt/PCP signaling pathway during fipronil induced lung inflammation. However, activation of the Wnt signaling pathway is the earliest stage of various types of oncogenesis [81] which subsequently results in Il23/Il17 driven tumor inflammation [82]. Our results indicate the role of *Il17c* to mediate the fipronil induced lung inflammation in Wnt/PCP signaling axis.

## Conclusion

We conclude that long-term exposure to low (4.75 mg kg^− 1^) and high (9.50 mg kg^− 1^) dose of fipronil alone or in combination with LPS alter the histoarchitecture and transcriptome profile of lungs. The data are significant because it describes the involvement of the PCP pathway triggered by *Wnt6* which leads to the downstream production of various cytokines during fipronil induced lung damage. We consider these data a platform for conducting further studies on the potential impact of fipronil and endotoxin interaction on other organs such as liver and kidney and subsequent use of mitigation strategies to control it.

## Additional files

Additional file 1: **Figure S1.** Heat map analysis of the commonly expressed gene in LPS (CL), high dose (1/10th of LD_50_) of fipronil alone (F10) or in combination with LPS (FL10) and a low dose (1/20th of LD_50_) of fipronil alone (F20) or in combination with LPS (FL20) group. **Figure S2-S4.** PCP pathway generated by IPA in LPS (CL), high dose (1/10th of LD_50_) of fipronil alone (F10) or in combination with LPS (FL10) and a low dose (1/20th of LD_50_) of fipronil alone (F20) or in combination with LPS (FL20) group. (ZIP 12216 kb)

Additional file 2: **Table S1.** Gene Ontology (GO) analysis of the enriched genes. (DOCX 14 kb)

## Abbreviations

c PLA2: Cellular phospholipase
DLC: Differential leukocyte count
GABA: Gamma Amino Butyric Acid
HMGB1: High mobility group box protein 1
IL: Interleukin
*Il17c*: Interleukin 17 C
*Il4*: Interleukin-4
Jnk: c-Jun N-terminal kinases
LD: Lethal Dose
LPS: Lipopolysaccharide
Map k8: Mitogen-activated protein kinase 8
mg: Milligram
PCP: Planar cell polarity
RNA: Ribonucleic Acid
TLR: Toll-Like Receptor
TNF: Tumor Necrosis Factor
*Wnt6*: Wingless-type MMTV integration site family, member 6
μl: Microliter
μM: Micromole
