# Supplementary figures and images for: Transcriptome analysis reveals the role of the PCP pathway in fipronil and endotoxin-induced lung damage
Source: Respir Res. 2019 Feb 1;20:24. doi: 10.1186/s12931-019-0986-1 (PMC6359862; doi:10.1186/s12931-019-0986-1)

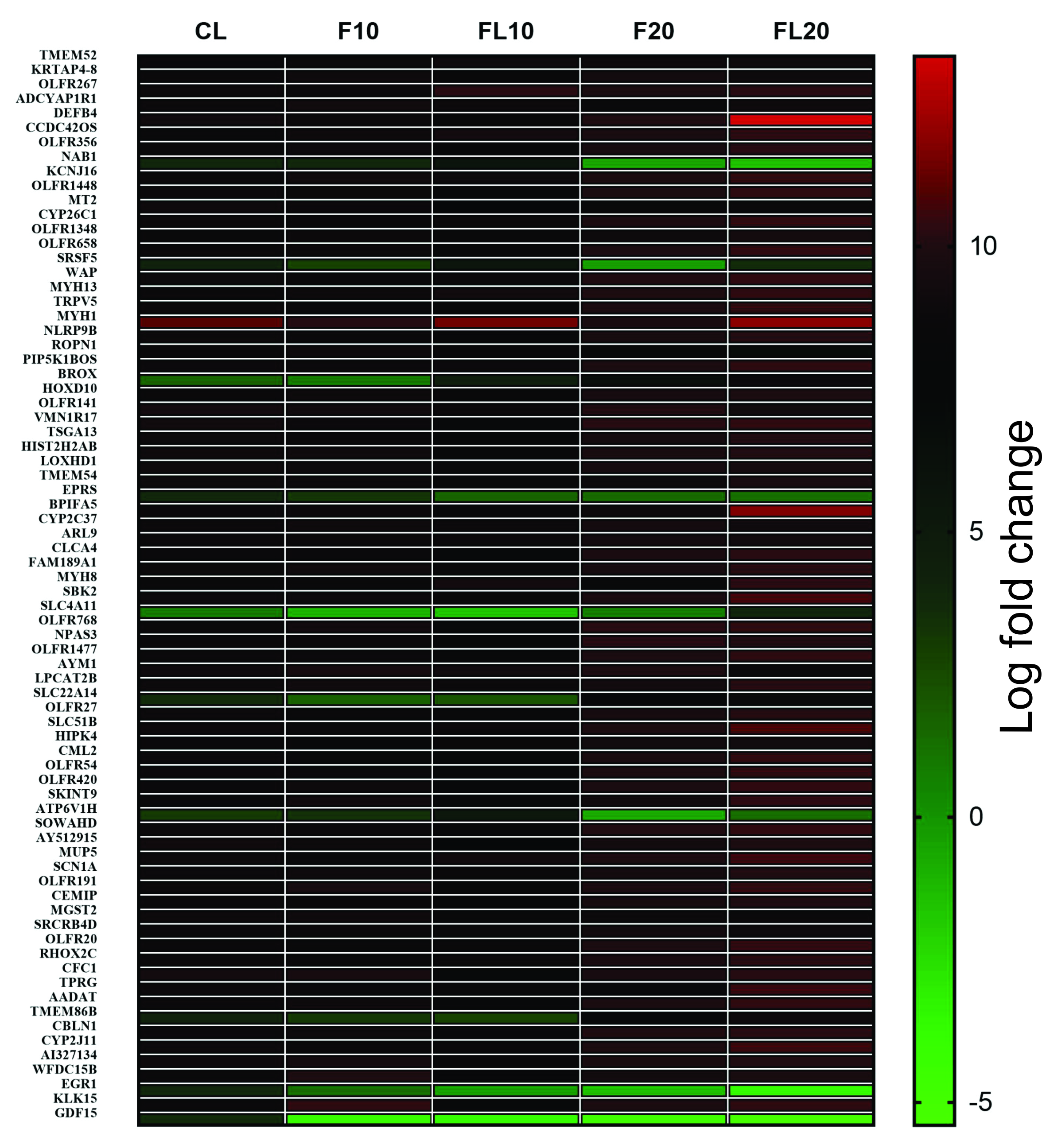

Supplement: Supplementary file 1 — Figure S1. Heat map analysis of the commonly expressed gene in LPS (CL), high dose (1/10th of LD50) of fipronil alone (F10) or in combination with LPS (FL10) and a low dose (1/20th of LD50) of fipronil alone (F20) or in combination with LPS (FL20) group. Figure S2-S4. PCP pathway generated by IPA in LPS (CL), high dose (1/10th of LD50) of fipronil alone (F10) or in combination with LPS (FL10) and a low dose (1/20th of LD50) of fipronil alone (F20) or in combination with LPS (FL20) group. (ZIP 12216 kb) [file 12931_2019_986_MOESM1_ESM.zip › S1.jpg]

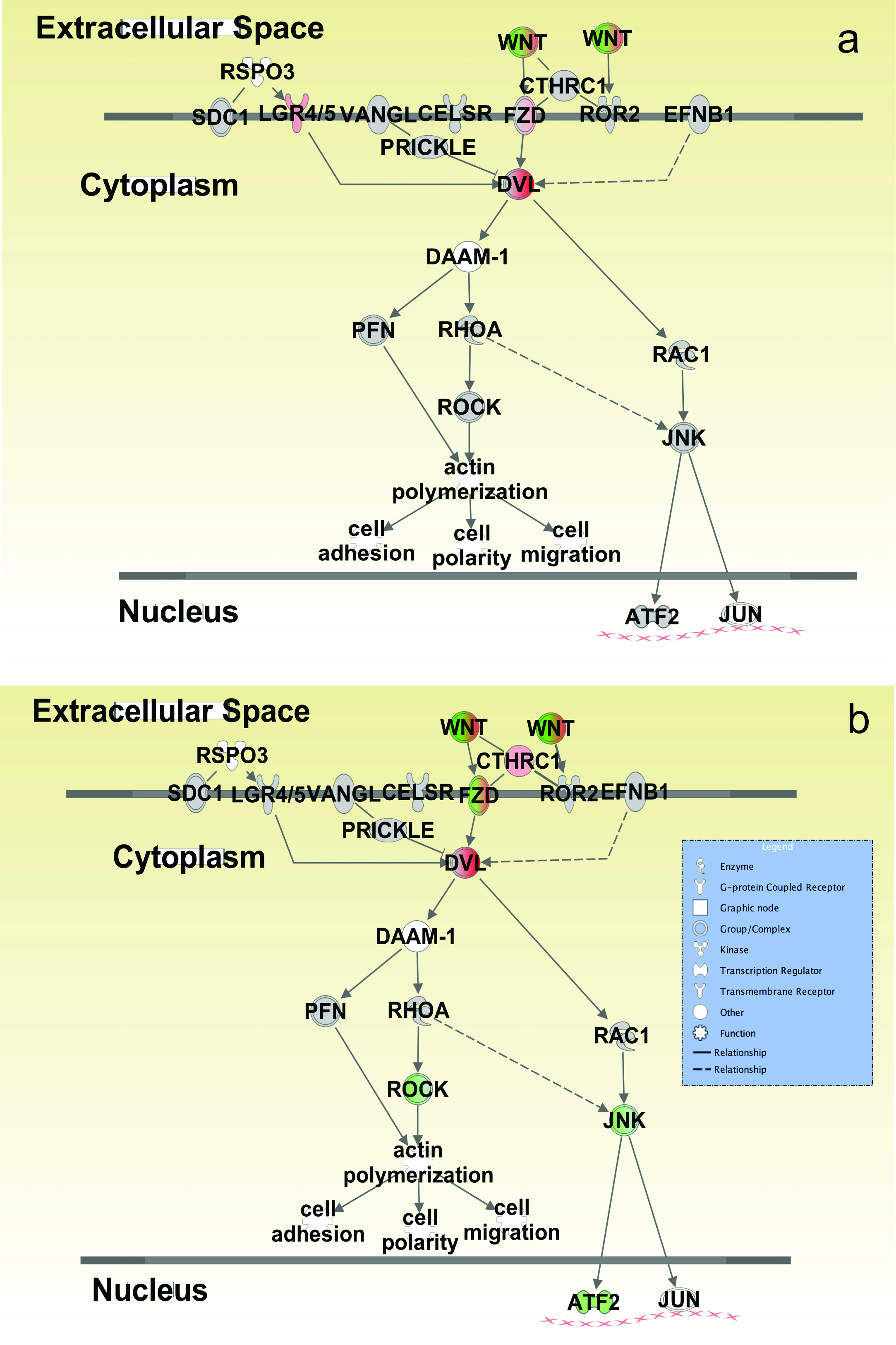

Supplement: Supplementary file 1 — Figure S1. Heat map analysis of the commonly expressed gene in LPS (CL), high dose (1/10th of LD50) of fipronil alone (F10) or in combination with LPS (FL10) and a low dose (1/20th of LD50) of fipronil alone (F20) or in combination with LPS (FL20) group. Figure S2-S4. PCP pathway generated by IPA in LPS (CL), high dose (1/10th of LD50) of fipronil alone (F10) or in combination with LPS (FL10) and a low dose (1/20th of LD50) of fipronil alone (F20) or in combination with LPS (FL20) group. (ZIP 12216 kb) [file 12931_2019_986_MOESM1_ESM.zip › S2.jpg]

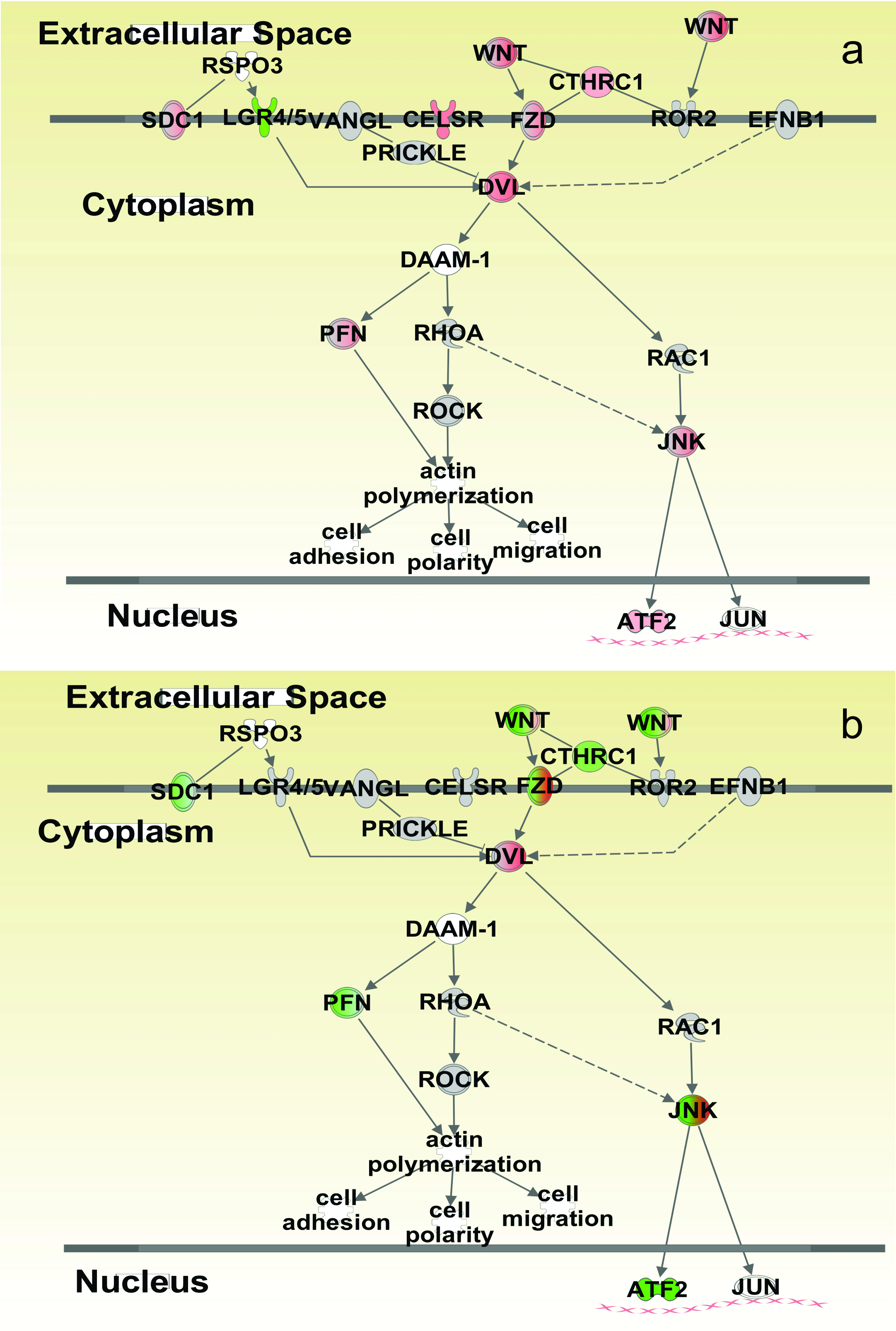

Supplement: Supplementary file 1 — Figure S1. Heat map analysis of the commonly expressed gene in LPS (CL), high dose (1/10th of LD50) of fipronil alone (F10) or in combination with LPS (FL10) and a low dose (1/20th of LD50) of fipronil alone (F20) or in combination with LPS (FL20) group. Figure S2-S4. PCP pathway generated by IPA in LPS (CL), high dose (1/10th of LD50) of fipronil alone (F10) or in combination with LPS (FL10) and a low dose (1/20th of LD50) of fipronil alone (F20) or in combination with LPS (FL20) group. (ZIP 12216 kb) [file 12931_2019_986_MOESM1_ESM.zip › S3.jpg]

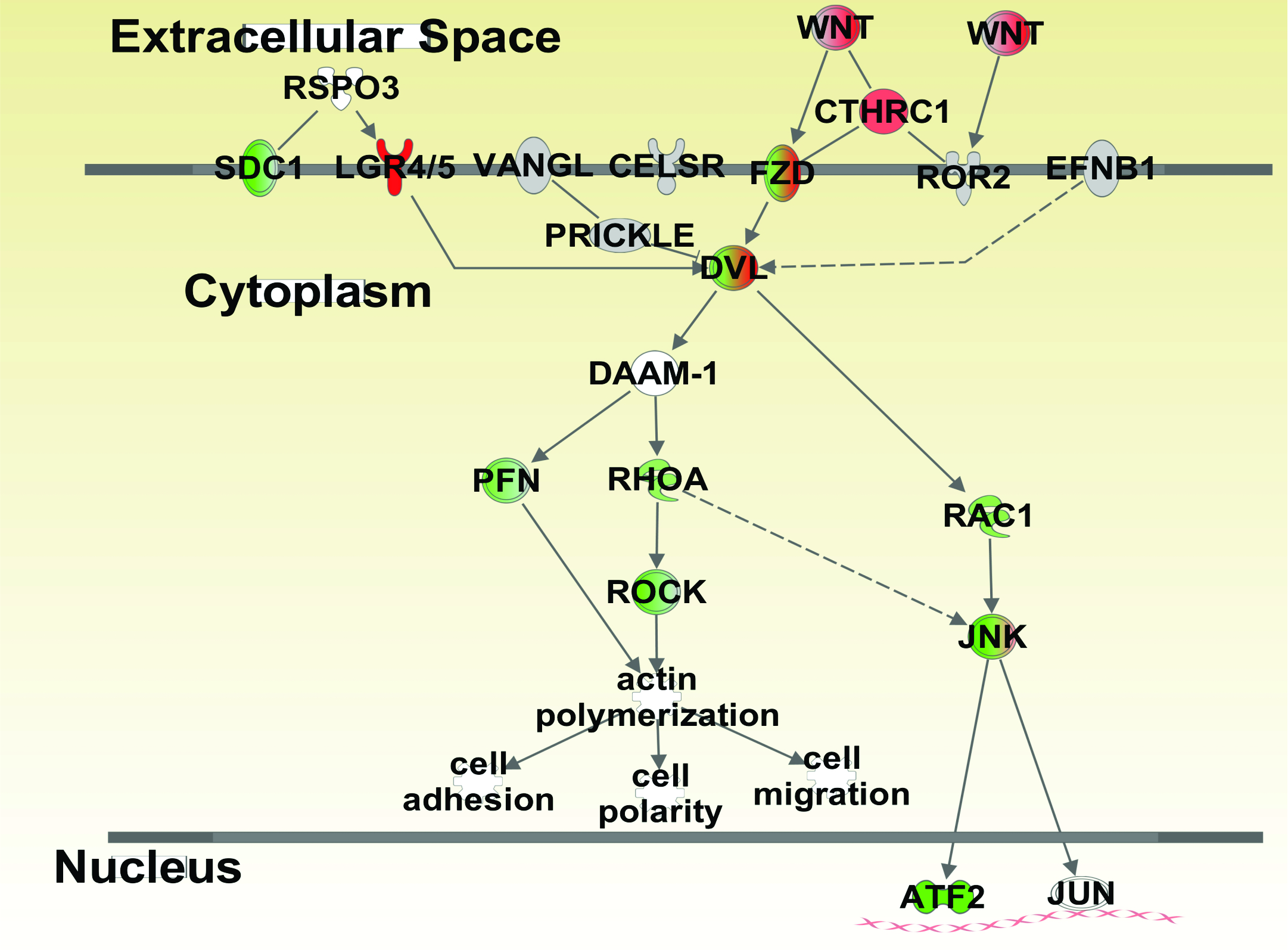

Supplement: Supplementary file 1 — Figure S1. Heat map analysis of the commonly expressed gene in LPS (CL), high dose (1/10th of LD50) of fipronil alone (F10) or in combination with LPS (FL10) and a low dose (1/20th of LD50) of fipronil alone (F20) or in combination with LPS (FL20) group. Figure S2-S4. PCP pathway generated by IPA in LPS (CL), high dose (1/10th of LD50) of fipronil alone (F10) or in combination with LPS (FL10) and a low dose (1/20th of LD50) of fipronil alone (F20) or in combination with LPS (FL20) group. (ZIP 12216 kb) [file 12931_2019_986_MOESM1_ESM.zip › S4.jpg]
