# Supplementary material for: Transcriptome analysis reveals the role of the PCP pathway in fipronil and endotoxin-induced lung damage
Source: Respir Res. 2019 Feb 1;20:24. doi: 10.1186/s12931-019-0986-1 (PMC6359862; doi:10.1186/s12931-019-0986-1)
Supplement: Supplementary file 2 — Table S1. Gene Ontology (GO) analysis of the enriched genes. (DOCX 14 kb) [file 12931_2019_986_MOESM2_ESM.docx]

| **GO ACCESSION** | **GO Term** | **Corrected p-value** | **Total genes** |
| --- | --- | --- | --- |
| GO:0003008 | system process | 3.03E-34 | 2492 |
| GO:0004871\|GO:0005062\|GO:0009369\|GO:0009370 | signal transducer activity | 4.42E-27 | 2299 |
| GO:0004872\|GO:0019041 | receptor activity | 0 | 2189 |
| GO:0004888\|GO:0004926 | transmembrane signaling receptor activity | 0 | 1917 |
| GO:0004930\|GO:0001622\|GO:0001623\|GO:0001624\|GO:0001625\|GO:0016526 | G-protein coupled receptor activity | 5.21E-13 | 627 |
| GO:0004984 | olfactory receptor activity | 0 | 1066 |
| GO:0005549 | odorant binding | 8.07E-19 | 449 |
| GO:0007186 | G-protein coupled receptor signaling pathway | 1.88E-41 | 1880 |
| GO:0007600 | sensory perception | 1.88E-41 | 1708 |
| GO:0007606 | sensory perception of chemical stimulus | 0 | 1340 |
| GO:0007608 | sensory perception of smell | 0 | 1096 |
| GO:0009593 | detection of chemical stimulus | 0 | 1154 |
| GO:0016021 | integral component of membrane | 1.49E-08 | 5636 |
| GO:0031224 | intrinsic component of membrane | 5.64E-08 | 5782 |
| GO:0038023 | signaling receptor activity | 0 | 2025 |
| GO:0050877 | nervous system process | 4.03E-36 | 2057 |
| GO:0050906 | detection of stimulus involved in sensory perception | 0 | 1191 |
| GO:0050907 | detection of chemical stimulus involved in sensory perception | 0 | 1127 |
| GO:0050911 | detection of chemical stimulus involved in sensory perception of smell | 0 | 1066 |
| GO:0051606 | detection of stimulus | 0 | 1284 |
| GO:0060089 | molecular transducer activity | 1.37E-38 | 2244 |
| GO:0099600 | transmembrane receptor activity | 0 | 1917 |
